# Existing and emerging strategies for fertility preservation in female patients with leukemia

**DOI:** 10.3389/fendo.2026.1778385

**Published:** 2026-06-01

**Authors:** Amirhossein Abazarikia, Amrita Purkayastha, Wonmi So, Mahsa Rasekhi, So-Youn Kim

**Affiliations:** 1 Department of Obstetrics, Gynecology and Reproductive Health, Rutgers New Jersey Medical School, Newark, NJ, United States; 2 Creighton University, Omaha, NE, United States; 3 Center for Immunity and Inflammation, Rutgers New Jersey Medical School, Newark, NJ, United States; 4 Center for Cell Signaling, Rutgers New Jersey Medical School, Newark, NJ, United States

**Keywords:** fertility preservation, infiltration, leukemia, ovary, treatment

## Abstract

Leukemia, the most common malignancy in childhood, affects individuals from prepubertal girl to women of reproductive age. It originates from immature hematopoietic cells in the bone marrow and disseminates systemically via the blood and lymphatic circulations. Although survival rates have improved substantially, standard treatments, including intensive chemotherapy and hematopoietic stem cell transplantation, often combined with total body irradiation, are highly gonadotoxic. Consequently, a significant proportion of survivors experience long-term endocrine dysfunction and infertility, with females at particular risk of primary ovarian insufficiency. Fertility preservation in leukemia patients presents significant challenges. In females, oocyte or embryo cryopreservation requires sexual maturity and ovarian stimulation, limiting its use in prepubertal girls. Ovarian tissue cryopreservation is therefore the primary option in this population. However, its application remains controversial due to the risk of leukemic cell contamination and potential disease reintroduction following transplantation. Experimental evidence indicates that leukemic cells can infiltrate reproductive tissues, potentially compromising tissue integrity and function. These findings underscore the need for careful evaluation of current approaches. This review synthesizes current clinical and experimental evidence, highlights key knowledge gaps, and discusses emerging strategies to improve the safety and efficacy of fertility preservation in female patients with leukemia.

## 1 Introduction

Leukemia is a group of hematologic malignancies characterized by clonal expansion of abnormal bone marrow cells that impair normal hematopoiesis and can disseminate systemically (1). Leukemias are broadly classified according to disease tempo (acute or chronic) and cellular lineage (lymphoid or myeloid), resulting in the major subtypes: acute lymphoblastic leukemia (ALL), chronic lymphocytic leukemia (CLL), acute myeloid leukemia (AML), and chronic myeloid leukemia (CML). Over the past several decades, advances in risk stratification, chemotherapy regimens, targeted therapies, and hematopoietic stem cell transplantation have dramatically improved survival outcomes for patients with leukemia, particularly among children and adolescents (2). Cure rates for pediatric ALL now approach 80-90%, and survival has also improved substantially in AML (3). In CML, the introduction of tyrosine kinase inhibitors transformed the disease from a fatal malignancy to a largely manageable chronic condition, with long-term survival exceeding 80% (4). Although CLL remains largely incurable, improved understanding of its molecular pathogenesis has led to more effective and durable treatment strategies. As a result, an increasing number of patients are surviving leukemia into adulthood (5–8).

Despite these therapeutic successes, leukemia remains a systemic disease with the capacity to infiltrate non-hematopoietic organs, both at initial diagnosis and at relapse (9). While extramedullary involvement has been reported across subtypes, particularly in CLL, the functional consequences of leukemic infiltration in peripheral organs are incompletely understood (10). Retrospective clinical studies have documented leukemic involvement in organs such as the spleen, liver, kidneys, adrenal glands, heart, and pancreas (11–16). In the spleen, for example, specialized macrophage populations within the red pulp have been shown to support leukemic stem cell survival and promote therapy resistance (17). Although extramedullary manifestations of AML are relatively uncommon, when present, they can involve multiple tissues and complicate or delay treatment initiation (18). Collectively, these observations underscore the systemic nature of leukemia and suggest that organ-specific complications, particularly during relapse, may be underrecognized.

In the United States alone, tens of thousands of new leukemia cases are diagnosed annually, with a disproportionate increase observed among children, adolescents, and young adults. As survival rates improve, long-term treatment-related toxicants have emerged as a major clinical concern. Among female survivors in this age group, fertility preservation consistently ranks among the most significant and unresolved quality-of-life issues.

The ovary contains the primordial follicle pool, which constitutes the ovarian reserve, a finite, non-renewable population of oocytes that determines reproductive lifespan and long-term endocrine function (19–21). Several clinical and experimental studies have reported leukemic cell infiltration into ovarian tissue, raising critical questions about how malignant cells interact with the ovarian microenvironment, disrupt follicular integrity, or accelerate depletion of the ovarian reserve (22, 23). Beyond direct ovarian damage, the ovary may also serve as a sanctuary site for residual leukemic cells, potentially contributing to disease persistence or relapse (24, 25). These concerns are particularly relevant in the context of fertility preservation strategies that involve ovarian tissue manipulation or transplantation (26).

In addition to the inherent risks posed by leukemia, standard treatments present a significant threat to female fertility. Chemotherapy and radiotherapy are well-established gonadotoxins that induce DNA damage and apoptosis in oocytes within primordial follicles, leading to irreversible depletion of the ovarian reserve. Because this reserve is nonrenewable, treatment-induced follicle loss can result in primary ovarian insufficiency (POI), long-term endocrine dysfunction, and infertility. Therefore, fertility preservation in patients with leukemia necessitates careful consideration of both the potential leukemic involvement of the ovary and the cytotoxic impact of anticancer therapies on the ovary. Chemotherapeutic agents target rapidly dividing cells, disrupting DNA integrity and key cellular processes such as replication and repair. These perturbations can trigger oocyte apoptosis, stromal fibrosis, vascular injury, or altered follicular differentiation. Elucidating these mechanisms of oocyte loss in leukemic patients provides critical insight into disease progression, informs optimal chemotherapy selection, and guides strategies to mitigate treatment-related reproductive complications.

This review focuses on female fertility preservation in the context of leukemia, with particular emphasis on ovarian function, leukemic infiltration, reproductive outcomes, and current preservation strategies across major leukemia subtypes, including ALL, CLL, AML, and CML. We integrate clinical observations, evidence from animal models, and mechanistic insights to elucidate how leukemia and its treatments, such as chemotherapy, radiotherapy, and hematopoietic stem cell transplantation, impact female reproductive potential. Additionally, we discuss existing and emerging fertility preservation approaches, including cryopreservation techniques and pharmacologic ovarian protection, evaluate potential biomarkers of ovarian reserve, and highlight critical knowledge gaps. By integrating recent advances in the field, this review aims to inform both oncologists and reproductive specialists and to support the development of safer and more effective fertility preservation strategies for female leukemia patients and survivors.

### 1.1 Leukemia: an overview

#### 1.1.1 Types

ALL arises from immature lymphoid progenitor cells and is characterized by the rapid proliferation of poorly differentiated blasts that lack normal immune function (27). Due to its aggressive clinical course, ALL progresses quickly and requires immediate treatment (7). It is the most common leukemia in childhood and is diagnosed across all age groups, with approximately 6,500 new cases reported annually in the United States (7). On a global scale, an estimated 487,000 new cases were reported in 2022, accounting for approximately 2–3% of all cancers worldwide (28). The majority of ALL cases (approximately 75-80%) are of B-cell origin, whereas 10-15% arise from T-cell precursors (29). T-cell ALL (T-ALL) tends to present at an older age and is often associated with a more aggressive disease course (29). B-cell ALL (B-ALL) can be further subdivided based on the presence or absence of the Philadelphia chromosome (Ph; BCR: ABL1). Historically, Ph-positive B-ALL has been associated with a poor prognosis (30). However, the introduction of BCR::ABL1 tyrosine kinase inhibitors has markedly improved outcomes, with current five-year survival rates approaching those observed in Ph-negative disease (31).

In contrast, CLL originates from more mature lymphoid cells and follows a relatively indolent clinical course. It is the most common leukemia in adults in Western countries (32). Although CLL remains largely incurable, many patients experience prolonged survival due to slow disease progression and significant advances in targeted therapies, including Bruton tyrosine kinase (BTK) and BCL-2 inhibitors, which have substantially improved disease control and quality of life (33). Additionally, the side effects of these therapies on reproductive organs are currently unknown. However, CLL primarily affects older adults, who are beyond reproductive age, and it is extremely rare in pediatric populations (34). This implies that gonadotoxicity is not usually a significant concern for patients with CLL.

AML is an aggressive hematologic malignancy arising from myeloid progenitor cells and is characterized by the rapid accumulation of immature myeloid blasts in the bone marrow and peripheral blood. AML occurs across the lifespan but is more common in adults, with incidence increasing with age (35). Despite advances in treatment, AML remains associated with significant morbidity and mortality, particularly in older patients (36). Long-term survival rates have improved in recent years, especially among younger patients, due to refinements in chemotherapy, stem cell transplantation, and the development of molecularly targeted therapies (37).

CML arises from relatively mature myeloid stem cells and is defined by the presence of the Philadelphia chromosome, which generates the BCR::ABL1 fusion oncogene (38). CML typically follows a slow, indolent course and is most frequently diagnosed in middle-aged and older adults. The advent of BCR::ABL1-targeted tyrosine kinase inhibitors has dramatically altered the natural history of the disease, transforming CML from a fatal malignancy into a manageable chronic condition for most patients, with long-term survival rates now exceeding 80% (39).

#### 1.1.2 Therapeutic approaches for leukemia and pathophysiological effects on the ovary

Therapeutic approaches for leukemia vary widely, ranging from intensive multi-agent chemotherapy and radiotherapy for acute leukemias to long-term targeted oral therapies for select chronic leukemias. While these treatments have substantially improved survival, many carry significant risks to reproductive health, with effects that may be immediate, delayed, or permanent (**Table 1**).

**Table 1:** Leukemia subtype, treatment, and fertility risk.

Leukemia type	Standard Treatment	Gonadotoxic Agents	Fertility Risk (Female)	Fertility Risk (Male)
B-ALL (Ph+)	TKIs + Chemotherapy/immunotherapy (e.g. blinatumomab) (39, 40)	TKIs, alkylators	Moderate to High	Variable
B-ALL (Ph-)	Multi-agent chemotherapy (hyper-CVAD), methotrexate, cytarabine (41)	Cyclophosphamide, Anthracyclines	High	Moderate to High
T-ALL	HSCT/Chemotherapy (Vincristine, corticosteroids, anthracycline, pegaspargase, nelarabine) (42–44)	Vincristine, anthracycline	Moderate	Unknown
AML	HSCT/Intensive chemotherapy (Cytarabine, anthracyclines, venetoclax, ivosidenib, azacitidine) (45, 46)	Cytarabine, Anthracyclines	Variable to High	Variable
CML	Long-term TKIs (47)	Chronic kinase inhibition	Low to Moderate (48–51)	Low
CLL	BTK, BCL2 inhibitors (52)	Limited data	Unknown	Unknown

B-ALL (Ph+), B-cell acute lymphoblastic leukemia with the Philadelphia chromosome-positive (Ph+); B-ALL (Ph-), B-cell acute lymphoblastic leukemia with the Philadelphia chromosome-negative (Ph-); T-ALL, T-cell acute lymphoblastic leukemia; AML, acute myeloid leukemia; CML, chronic myeloid leukemia; CLL, chronic lymphocytic leukemia; TKIs, tyrosine kinase inhibitors; CVAD, Cyclophosphamide, Vincristine (Oncovin), Adriamycin (doxorubicin), and Dexamethasone; HSCT, hematopoietic stem cell transplantation; BTK, Bruton’s tyrosine kinase; BCL2, B-cell lymphoma 2.

ALL occurs predominantly in pediatric and young patients, whereas AML is more common in older adults, although it can also affect younger populations. Most treatment protocols for ALL involve relatively lower doses of chemotherapy that are not typically associated with severe gonadal toxicity. In AML patients, the use of alkylating agents is often limited in many regimens, and therefore fertility is generally less severely affected (40, 53). Nonetheless, adolescent and young adult female leukemia patients are at a significantly higher risk of infertility compared with the non-cancer population. Based on data from a cohort study of 292 leukemia patients, infertility was detected in 40 individuals, corresponding to a prevalence of 13.7%. When compared with the average infertility rate reported among survivors of various other cancers (approximately 11.5%), this rate remains higher than the overall cancer survivor average (54).

In Ph-positive B-ALL, standard treatment includes BCR::ABL1 tyrosine kinase inhibitors (TKIs) in combination with multi-agent chemotherapy or immunotherapy, such as blinatumomab (48, 49). In addition to inhibiting BCR::ABL1, TKIs also target kinases such as c-KIT and platelet-derived growth factor receptor (PDGFR), which play essential roles in folliculogenesis and ovarian stromal function (50, 51). The reproductive toxicity of TKIs has been most extensively characterized in CML (55), where agents, particularly imatinib, have been associated with amenorrhea, menstrual irregularities, and reduced anti-Müllerian hormone (AMH) levels, consistent with diminished ovarian reserve (41). Nonetheless, the impact of tyrosine kinase inhibitors (TKIs) on ovarian reserve appears to be reversible after treatment discontinuation. Evidence indicates increases in AMH levels and antral follicle counts following cessation of TKI therapy (42, 43), suggesting that TKI-associated ovarian dysfunction is likely minimal and transient. Although serum AMH is widely used as a marker of ovarian reserve, it does not precisely reflect ovarian reserve and exhibits substantial inter-individual variability. Therefore, the effects of TKIs on ovarian reserve and long-term reproductive potential in leukemia survivors remain inconclusive.

For Philadelphia chromosome-negative (Ph-) B-ALL, different chemotherapy remains the cornerstone of treatment, particularly in pediatric patients. Adult regimens often include hyper-CVAD, which is also used in certain non-Hodgkin lymphomas (44). This intensive protocol alternates between Cycle A (cyclophosphamide, vincristine, doxorubicin, and dexamethasone) and Cycle B (methotrexate and cytarabine), exposing patients to multiple gonadotoxic agents administered concurrently or sequentially (45).

T-cell acute lymphoblastic leukemia (T-ALL) is treated with combination chemotherapy regimens that typically include pegylated asparaginase (46). Early T-cell precursor ALL is considered a high-risk subtype, for which hematopoietic stem cell transplantation (HSCT) may be recommended. Treatment is generally structured into three phases: induction (vincristine, corticosteroids, anthracycline, and pegaspargase), consolidation (intensified chemotherapy with or without HSCT), and maintenance (prolonged low-dose chemotherapy, with nelarabine added in select cases) (47, 56).

Patients diagnosed with AML are typically treated with chemotherapy combined with HSCT to eliminate fast-growing cancer cells in the blood and bone marrow (57). The common chemotherapy drugs used for AML include cytarabine, anthracyclines such as daunorubicin or idarubicin, and other agents like mitoxantrone, azacitidine, decitabine, and cladribine. Targeted therapies have improved outcomes in specific patients. In older adults, venetoclax with a hypomethylating agent is standard first-line therapy, with ivosidenib plus azacitidine for IDH1-mutant cases. Venetoclax is also being tested in younger patients, showing promising early results (52).

In CML, first-line therapy consists of TKIs such as imatinib, dasatinib, nilotinib, or bosutinib (58). However, because TKI treatment in CML is often prolonged or lifelong, concerns regarding cumulative reproductive toxicity are particularly relevant for patients of reproductive age (59).

For CLL, traditional chemoimmunotherapy has largely been replaced by targeted agents and is now used only in select young and fit patients with favorable molecular features, such as mutated IGHV (60). Contemporary treatment strategies primarily rely on Bruton’s tyrosine kinase (BTK) inhibitors (e.g., ibrutinib) or the B-cell lymphoma 2 (BCL2) inhibitor venetoclax, often combined with anti-CD20 monoclonal antibodies such as obinutuzumab (61).

However, the direct effects of all these agents on ovarian function have not been extensively studied.

##### 1.1.1.1 Pathophysiological effects of chemotherapeutic agents on the ovary

Over the past decades, the development of chemotherapeutic agents such as cyclophosphamide, busulfan, and other gonadotoxic agents, along with advances in bone marrow transplantation, which consists of intensive chemotherapy combined with/without total body irradiation (TBI), has significantly improved treatment outcomes for childhood acute leukemia (62). Across leukemia subtypes, treatment-related fertility risk varies substantially depending on the therapeutic regimen (**Table 1**). Regimens for acute leukemias are often associated with a high risk of POI due to their detrimental impact on ovarian reserve (**Table 2**) (19).

**Table 2:** Pathophysiological effects of gonadotoxic leukemia therapies on the ovary.

Therapeutic agent/modality	Primary mechanism of damage	Cellular/molecular targets	Ovarian pathophysiological phenotype	Clinical outcome
Cyclophosphamide (alkylating agent)	DNA crosslinking → double-strand breaks → apoptosis	Oocytes (primordial follicles) and granulosa cells, Activation of TAp63, PUMA/NOXA pathways (63–68)	Rapid depletion of primordial follicles, Fibrosis, increased follicular atresia, reduced follicle size, vascular damage (61, 62)	Diminished ovarian reserve, POI, infertility
Busulfan (alkylating agent)	DNA alkylation → cytotoxicity	Primordial follicles, stromal cells	Severe follicle loss, stromal damage, synergistic toxicity with cyclophosphamide (69)	High risk of permanent infertility, especially in conditioning regimens (70)
Anthracyclines (e.g., doxorubicin)	Oxidative stress, DNA intercalation, mitochondrial damage	Granulosa cells, stromal cells	Increased apoptosis, impaired follicle support, stromal injury	Reduced ovarian reserve, subfertility (71)
Cytarabine (low gonadotoxicity)	Antimetabolite → Inhibition of DNA synthesis	Rapidly dividing cells (limited effect on dormant follicles)	Minimal direct follicular damage (alone)	Low fertility risk unless combined with other agents (71)
Combination chemotherapy (e.g., hyper-CVAD)	Additive/synergistic DNA damage and cytotoxicity	Multiple ovarian compartments	Accelerated follicle depletion, increased atresia, impaired folliculogenesis	Moderate-high infertility risk (72)
Total Body Irradiation (TBI)	Ionizing radiation → DNA double-strand breaks, oxidative stress (73)	Oocytes (highly radiosensitive), stromal and vascular cells	Massive primordial follicle loss, cortical fibrosis, vascular damage, ovarian atrophy (73, 74)	Near-complete ovarian failure at high doses (>10–15 Gy) (73, 75)
Hematopoietic stem cell transplantation (HSCT) conditioning with chemotherapy and/or TBI	DNA alkylation → cytotoxicity, Ionizing radiation → DNA double-strand breaks, oxidative stress	Entire ovarian tissue (oocytes, stroma, vasculature)	Follicle depletion, fibrosis, vascular compromise	Very high risk of POI and permanent infertility (76–80)
Busulfan + Cyclophosphamide (conditioning regimen)	Synergistic DNA damage and cytotoxicity	Oocytes, stromal and vascular compartments	Severe ovarian destruction, profound follicle depletion, structural damage	Extremely high risk of POI (especially in postpubertal females) (81)

CVAD, Cyclophosphamide, Vincristine (Oncovin), Adriamycin (doxorubicin), and Dexamethasone; HSCT, hematopoietic stem cell transplantation; POI, Primary ovary insufficiency; PUMA, p53 upregulated modulator of apoptosis; Gy, Gray.

Histological analyses of ovarian morphology in childhood ALL survivors demonstrated a clear reduction in follicle numbers, cortical fibrosis, and vascular damage following treatment (40). Interestingly, the severity of these changes was age-dependent, becoming more pronounced with increasing age (70). In another study, histological analyses of ovarian tissue from chemotherapy-treated and untreated patients were performed. The results showed that the density of atretic primordial follicles was higher in the treated group, and intact primordial follicles were smaller than those in untreated patients (69).

In a nationwide survey that included 1,476 leukemia survivors, the results of fertility outcomes indicated that 104 leukemia patients (about 7%) had exhibited infertility. These data demonstrate that a substantial proportion of leukemia cancer survivors experience infertility due to the treatment, underscoring declining fertility as a significant long-term concern with important implications for quality of life (71). The risk of gonadal failure is much higher when multiple chemotherapeutic agents are used in combination (82). In one study, different criteria, including pubertal development, were evaluated to assess the side effects of conditioning regimens with and without TBI before BMT in AML patients. Follow-up results indicated that two girls in the busulfan-cyclophosphamide-treated group showed POI characterized by delayed puberty and menopausal levels of luteinizing hormone (LH) and follicle-stimulating hormone (FSH), and both required long-term hormonal replacement therapy (83).

##### 1.1.1.2 Pathophysiological effects of total body irradiation on the ovary

In leukemia patients at high risk of relapse or with recurrent disease following chemotherapy, bone marrow transplantation (BMT) is an effective strategy to prevent relapse and prolong disease-free survival (DFS), particularly in children with AML in first remission (84). Myeloablative conditioning regimens used prior to transplantation typically consist of either TBI combined with an alkylating agent or two alkylating agents administered at myeloablative doses (85). TBI delivers ionizing radiation uniformly across the entire body and is commonly incorporated into conditioning regimens for patients with high-risk or relapsed leukemia (86, 87). Despite the use of fractionated schedules to mitigate systemic toxicity, the ovaries are typically not shielded and remain exposed to substantial radiation, rendering them highly susceptible to damage (72). Conditioning regimens that include high-dose alkylating agents and/or TBI are associated with a significant risk of permanent infertility. The degree of gonadotoxicity depends on both the radiation dose (Gy) and the specific chemotherapeutic agents used, particularly in combination with cyclophosphamide. The radiation dose required to induce prolonged amenorrhea is approximately >15 Gy in prepubertal girls and >10 Gy in postpubertal girls; however, doses exceeding 10 Gy are frequently used in clinical protocols. In addition, the use of busulfan in combination with cyclophosphamide in certain regimens further exacerbates gonadal toxicity (81).

The ovarian follicle pool is exquisitely sensitive to ionizing radiation, and cumulative ovarian doses administered during TBI are sufficient to cause profound and often irreversible follicular depletion, fibrosis, vascular damage, and ovarian atrophy (81, 88). Even with fractionated radiation, ovarian damage persists, as primordial follicles are sensitive to radiation, leading to DNA damage and depletion due to radiation-induced double-strand breaks (81). Clinical and epidemiological studies consistently demonstrate a strong dose-dependent relationship between ovarian radiation exposure and permanent loss of reproductive function. Nearly all women receiving ovarian doses exceeding 20 Gy develop POI, while the majority of those exposed to doses above 10 Gy also experience irreversible infertility (76). Although younger age at exposure may confer limited protection due to a larger initial follicle reserve, TBI-associated ovarian failure has been reported across all age groups, underscoring the high sterilizing potential of this treatment modality.

##### 1.1.1.3 Pathophysiological effects of localized radiation therapy on the ovary

Localized radiation therapy involves delivering ionizing radiation to a defined anatomical region or target organ, rather than exposing the entire body. In the treatment of leukemia, the most common historical application of this approach has been cranial irradiation, particularly in patients with ALL (89). In some cases, craniospinal irradiation (CSI) has also been used when central nervous system involvement is present (89). Because cranial and CSI fields are anatomically confined to the head, and the spine and the ovaries remain outside the primary radiation field (73, 90, 91). Cranial-only irradiation is generally limited to low-level scattered radiation, often estimated to be below 1 Gy depending on factors such as treatment geometry, field size, and shielding. Typically, in the treatment of Acute Lymphoblastic Leukemia (ALL), prophylactic cranial irradiation is administered at doses of 18–24 Gy to the brain. At these dose levels, direct damage to the ovarian follicle reserve is rare because the ovaries are outside the radiation field. Consequently, any adverse reproductive effects are thought to arise primarily from disruption of the hypothalamus-pituitary axis rather than from direct ovarian irradiation (74, 75, 92). The risk to ovarian reserve in these patients, therefore, arises primarily from indirect mechanisms, including prior or concurrent exposure to gonadotoxic chemotherapy and radiation-induced injury to central neuroendocrine structures. However, higher doses of cranial irradiation, particularly above 30 Gy, can disrupt hypothalamic or pituitary function, leading to reduced gonadotropin secretion and secondary ovarian failure. Thus, while localized cranial radiation minimizes direct ovarian damage, its endocrine consequences remain an important consideration in long-term reproductive outcomes.

##### 1.1.1.4 Pathophysiological effects of hematopoietic stem cell transplantation on the ovary

Hematopoietic stem cell transplantation (HSCT) is an established therapeutic approach with curative or consolidative potential and is widely applied in patients with both malignant and non-malignant conditions. The primary indications for HSCT include hematological malignancies such as leukemia, lymphoma, and multiple myeloma, as well as non-malignant hematological disorders, including aplastic anemia, thalassemia, and Fanconi’s anemia, among others (93).

Despite its therapeutic benefits, HSCT, particularly when preceded by intensive conditioning regimens incorporating high-dose chemotherapy and/or TBI, is associated with profound gonadotoxicity (85). Among treatment modalities for leukemia, HSCT conditioning is considered the most damaging to ovarian function and frequently leads to irreversible infertility. HSCT represents one of the highest-risk interventions for reproductive failure (94). Thus, fertility preservation counseling and intervention should be prioritized prior to initiation of transplant-based therapy. A substantial proportion of girls undergoing HSCT develop acute ovarian insufficiency during or shortly after treatment, often manifesting as primary amenorrhea and failure to initiate pubertal development (95). A recent study of women who underwent HSCT for leukemia prior to puberty reported that, among 178 patients, 106 (60%) required pubertal induction with hormone replacement therapy, whereas 72 (40%) experienced spontaneous menarche. Importantly, even among those who experienced spontaneous pubertal onset, nearly half subsequently developed POI (46%), most commonly within the first five years following transplantation (96).

Age at the time of HSCT has emerged as a critical determinant of ovarian outcomes. Younger patients demonstrate a higher likelihood of spontaneous pubertal development and sustained ovarian function, whereas transplantation during later childhood or early adolescence is associated with a markedly increased need for pubertal induction and a higher risk of permanent ovarian failure (97). More than 65% of patients who underwent HSCT before 4.8 years of age experienced spontaneous menarche, and nearly 50% had not developed POI at the time of last follow-up. In contrast, over 85% of patients who underwent HSCT after 10.9 years of age did not achieve spontaneous menarche and required pubertal induction with hormone replacement therapy (97). Although spontaneous pregnancies have been reported following childhood HSCT, they remain relatively uncommon, underscoring the limited preservation of reproductive potential after treatment.

### 1.2 Direct effects of leukemia on female reproductive organs

#### 1.2.1 Leukemic cell infiltration

##### 1.2.1.1 Ovary

Because leukemia originates in the bone marrow and malignant cells circulate systemically, there is a substantial risk of leukemic contamination in ovarian cortical fragments and whole ovarian tissue (22). This concern is particularly relevant for fertility preservation strategies involving ovarian tissue cryopreservation (OTC), as circulating leukemic cells may infiltrate the ovary prior to tissue harvesting. Accordingly, several studies have investigated the presence of leukemic cells in ovarian tissue obtained from patients with leukemia and from animal models (77, 78, 98, 99).

In another clinical study, young women with acute leukemia who underwent ovarian tissue transplantation after achieving remission and receiving allogeneic HSCT reported no cases of disease recurrence attributable to the graft. The apparent safety of this approach may reflect effective leukemic clearance achieved by induction regimens that include relatively non-gonadotoxic agents, such as cytarabine and daunorubicin, administered prior to OTC. Nevertheless, these observations are limited to selected patient populations and may not be generalizable across leukemia subtypes or treatment protocols (79).

Experimental studies further support the concept of ovarian involvement in leukemia (80). In murine models of AML, leukemic cells have been shown to infiltrate ovarian tissue, particularly within the stromal compartment and among the granulosa cells of antral follicles. These findings provide compelling evidence that leukemic cells can access and persist within the ovary; however, the direct functional consequences of such infiltration on folliculogenesis, oocyte quality, and long-term ovarian reserve remain poorly defined (99).

##### 1.2.1.2 Other reproductive organs (uterus, vagina, cervix)

In addition to the ovaries, other female reproductive organs, including the uterus, cervix, and vagina, play essential roles in reproductive function and overall reproductive health. The uterus serves as the site for embryo implantation and fetal development, while the cervix functions as a gateway between the uterus and the vaginal canal, providing both protective and supportive roles. The vagina, as the distal component of the reproductive tract, facilitates sperm transport, serves as the birth canal during delivery, and contributes to the maintenance of the reproductive tract’s microbial and immunological environment. Together, these organs coordinate complex physiological processes critical to successful reproduction.

In patients with leukemia, these reproductive tissues may also be susceptible to leukemic involvement due to the systemic nature of the disease. Clinical reports have documented extramedullary infiltration of leukemic cells in uterine, cervical, and vaginal tissues, most often identified at autopsy or during evaluation for abnormal gynecologic symptoms (100, 101). Such infiltration may disrupt tissue architecture, impair endometrial receptivity, and alter local immune signaling, potentially compromising reproductive capacity (102).

In addition to direct leukemic infiltration, these organs are highly sensitive to the indirect effects of leukemia therapy. Chemotherapy and radiotherapy can induce uterine vascular injury, stromal fibrosis, and endometrial thinning, changes that may persist long after treatment completion and negatively affect implantation and pregnancy outcomes (103). Cervical and vaginal tissues may also be affected, with treatment-associated mucosal atrophy, reduced elasticity, and altered microbiota contributing to sexual dysfunction and reproductive morbidity (104, 105).

Although less extensively studied than ovarian toxicity, leukemia- and treatment-related alterations in the uterus, cervix, and vagina represent important yet underrecognized contributors to impaired reproductive outcomes in female leukemia survivors. Further research is needed to define the prevalence, mechanisms, and long-term clinical consequences of leukemic involvement in these tissues and to inform fertility preservation strategies that address the entire reproductive tract, rather than the ovary alone.

#### 1.2.2 Changes in the gonadal microenvironment

Leukemic cells can alter the ovarian microenvironment by promoting a pro-inflammatory state through cytokine signaling. In a murine model of AML, ovarian infiltration by leukemic cells was associated with increased levels of inflammatory mediators, including tumor necrosis factor alpha (TNF-α) and cyclooxygenase-2 (COX-2), within ovarian tissue (99). TNF-α is known to disrupt normal ovarian function. Experimental exposure of rodent ovaries to TNF-α reduces ovulated oocyte numbers and leads to unruptured follicles, with granulosa cells undergoing apoptosis (106). These findings suggest that leukemia-driven TNF-α elevation may impair ovulation and promote follicular atresia. Supporting this, AML-infiltrated ovaries exhibited a marked reduction or absence of corpora lutea, indicating compromised ovulatory activity (99). In addition to systemic inflammatory signals, the ovary itself produces cytokines such as leukemia inhibitory factor (LIF), which are critical for follicle regulation (107). Infiltration by leukemic cells may disrupt this local cytokine balance (99). Collectively, these alterations indicate that chronic inflammatory signaling induced by leukemic cells can reshape the ovarian microenvironment, potentially impairing follicle development, ovulation, and accelerating follicle depletion.

### 1.3 Mechanisms of ovarian damage induced by leukemia therapies

The ovaries are highly sensitive to DNA damage induced by gonadotoxicity, such as chemotherapy or radiation therapy (108). Oocytes in primordial follicles are non-dividing cells arrested in a prolonged prophase I. However, they actively respond to DNA damage by initiating either repair or apoptotic mechanisms (108). Recent studies have demonstrated that irradiation and chemotherapeutic agents trigger DNA damage-induced apoptotic pathways in oocytes (88, 108–113). Alkylating agents, such as cyclophosphamide and busulfan, damage DNA and induce apoptosis in oocytes, leading to the depletion of primordial follicles, fibrosis, and vascular damage (62, 69–71). However, anthracyclines, such as doxorubicin, and cytarabine exhibit low gonadotoxicity because they primarily damage growing follicles by affecting rapidly dividing granulosa cells (82).

**Figure 1:**
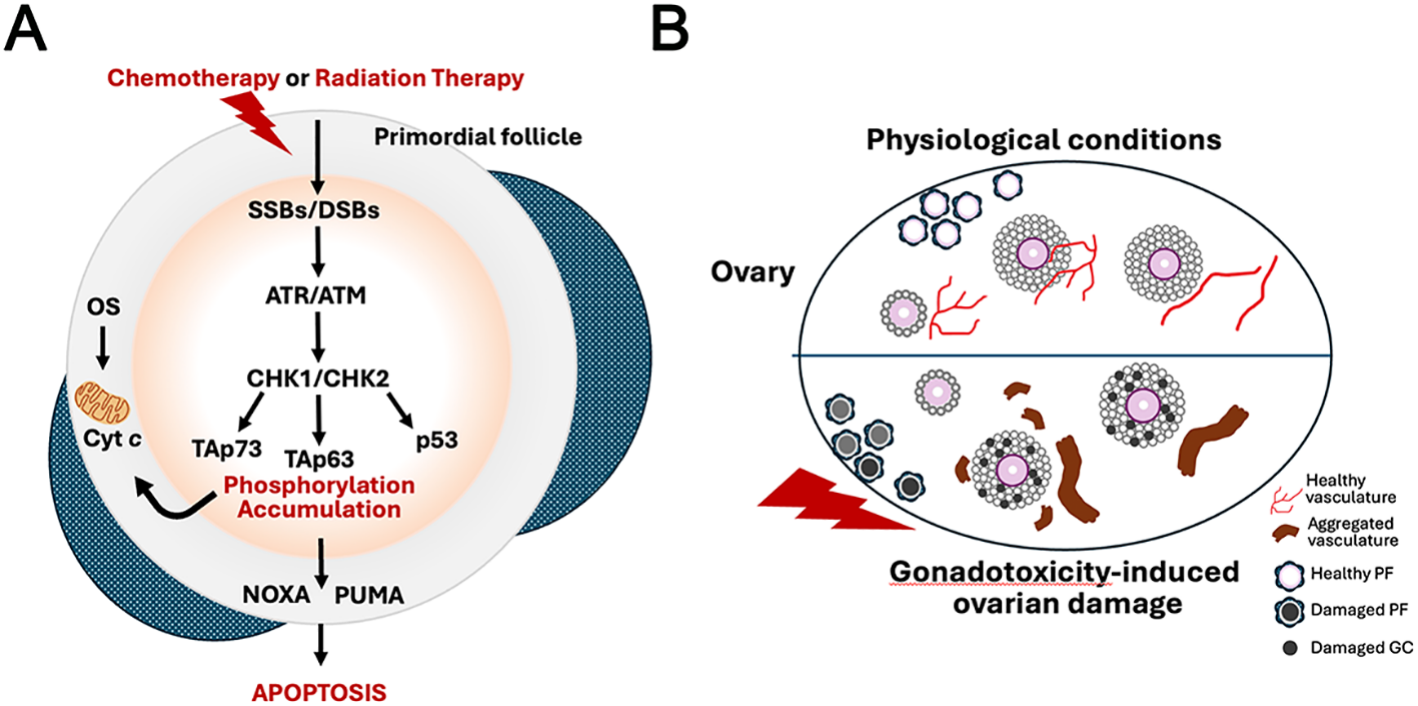
Molecular and cellular mechanisms of chemotherapy/radiation-induced ovarian damage and gonadotoxicity. **(A)** Schematic illustrating the DNA damage response pathway leading to apoptosis in oocytes within primordial follicles following exposure to chemotherapy or radiation therapy. These treatments induce single-strand breaks (SSBs) or double-strand breaks (DSBs) in oocyte DNA. The damage is sensed by ATR and/or ATM kinases, which activate checkpoint kinases CHK1 and/or CHK2. This leads to phosphorylation and accumulation of the p53 family members TAp73 and TAp63 (with p53 also involved). Activated TAp63/TAp73 upregulate the pro-apoptotic BH3-only proteins NOXA and PUMA, triggering caspase-dependent apoptosis in the oocyte. TAp63 signaling induces mitochondrial outer membrane permeabilization, leading to cytochrome c (Cyt. c) release and ultimately oocyte apoptosis. Oxidative stress (OS) can further contribute by promoting cytochrome c release. The pathway leads to depletion of the primordial follicle reserve, a major cause of gonadotoxicity and primary ovarian insufficiency. **(B)** A diagram illustrating ovarian damage caused by gonadotoxicity, with the ovary shown at the bottom. The upper panel shows a healthy ovary with abundant healthy primordial follicles (PF; light pink), intact granulosa cells (GC), and a well-organized, branched healthy vasculature (red). The lower panel illustrates damage caused by gonadotoxic agents (e.g., chemotherapy or radiation therapy), featuring damaged primordial follicles (dark/gray PFs), damaged granulosa cells (dark GCs), and pathological vascular changes, including aggregated or disrupted vasculature (brown structures). This leads to overall ovarian damage, reduced follicle pool, impaired vascular support, and compromised reproductive function.

At the molecular level, this damage is mediated through activation of the DNA damage checkpoint in oocytes, leading to stabilization of TAp63, a p63 isoform specifically expressed in oocytes within primordial follicles (63, 88, 114–116). TAp63, when activated by phosphorylation, initiates a cascade of signaling pathways that leads to apoptosis (88). Mouse studies have identified the BCL-2 family members PUMA and NOXA as critical downstream effectors that execute oocyte apoptosis in response to gonadotoxic DNA damage (**Figure 1A**) (117). In addition to direct effects on oocytes, gonadotoxic agents can also damage the ovarian stroma and vasculature, further compromising ovarian function (**Figure 1B**) (118).

### 1.4 Patient age and pubertal status in fertility outcomes

Patient age and pubertal status at the time of leukemia diagnosis are critical determinants of both treatment tolerance and long-term reproductive outcomes. Pediatric patients with ALL generally experience superior survival outcomes compared with adults, regardless of treatment modality. Children also demonstrate greater tolerance to intensive chemotherapy, whereas treatment of adult ALL is more frequently complicated by toxicity-related dose interruptions, treatment delays, or premature discontinuation, which can adversely affect outcomes (40, 64).

Despite improved survival in pediatric populations, data on long-term fertility and pregnancy outcomes following leukemia treatment remain limited. Notably, available evidence is more robust for women diagnosed with leukemia during childhood than for those diagnosed in adulthood, largely reflecting the higher survival rates among pediatric patients (64). Analyses from the Childhood Cancer Survivor Study have shown that female survivors treated with chemotherapy have a significantly lower likelihood of achieving a live birth compared with their healthy siblings, highlighting the lasting reproductive consequences of early-life cancer therapy (65).

Assessment of ovarian reserve in premenopausal women treated with chemotherapy alone for acute leukemia, without subsequent hematopoietic stem cell transplantation, suggests subclinical reproductive impairment (66). Although some studies have not identified statistically significant differences in ovarian reserve markers when compared with age-matched controls, a consistent trend toward reduced ovarian reserve has been observed among leukemia survivors (67, 68). These findings suggest that even in the absence of overt ovarian failure, chemotherapy exposure during childhood or adolescence may result in diminished reproductive potential later in life.

It is important to note psychosocial and ethical challenges with prepubertal patients. Since they are minors, they cannot make fully autonomous decisions regarding their future fertility. The 2025 ASCO Fertility Preservation Guideline (119) Update states that parents or guardians must make decisions on behalf of their children. A significant ethical dilemma arises in obtaining pediatric patient assent and parental consent for procedures that may not yield benefits for many years and carry associated risks (120). Given the complexity of consenting to this procedure, a two-stage consent process can be utilized. The first stage occurs at the time of diagnosis when the decision to harvest and store tissue is made. The second stage takes place after treatment, at an age appropriate for development, when the decision about whether and how to use the stored material is considered (121). This approach ensures that the patient is not obligated to use the samples in the future. To combat the emotional impact of fertility discussions on younger patients, the NCCN AYA Oncology Guidelines recommend referral to psychosocial support services and financial counseling (National Comprehensive Cancer Network. 2026, February 27). Finally, health care providers should take a patient centered approach and ensure that decisions are driven by the patient’s best interest.

### 1.5 Fertility preservation strategies for female leukemia patients

As previously noted, completion of therapy can lead to infertility in cancer survivors who are within their reproductive age (64, 122). In response, multiple professional societies have established guidelines outlining recommended fertility preservation strategies that should be implemented prior to the initiation of treatment. A summary of these recommendations is provided below.

#### 1.5.1 Oocyte and embryo cryopreservation

Ovarian stimulation for oocyte and embryo vitrification is considered a first-line treatment for fertility preservation in adult women (123). The success of this method depends on several factors, including the patient’s age, baseline ovarian reserve, and individual responsiveness to exogenous gonadotropin stimulation (124). Oocyte cryopreservation is an optimal fertility preservation strategy, as it does not require surgery and carries no risk of malignant cell contamination (125). This approach is particularly advantageous for young cancer patients who are single and prefer not to use donor sperm (125). Data on the efficacy of oocyte cryopreservation in oncology patients remain limited due to several factors, including its more recent adoption compared to embryo cryopreservation and the relatively low rate of patients returning to use their cryopreserved oocytes (123). Nevertheless, available evidence indicates that the efficacy of oocyte cryopreservation in cancer patients does not differ significantly from that observed in non-cancer populations (126). Importantly, success rates correlate positively with the number of oocytes retrieved. Therefore, in cancer patients who are unable to complete an ovarian stimulation cycle before treatment, or whose ovaries exhibit a poor response to stimulation, the likelihood of achieving a successful outcome is reduced (127, 128).

Embryo cryopreservation is regarded as the gold standard among fertility preservation techniques, particularly for patients who have a male partner or choose to use donor sperm. This method enables long-term preservation of embryos with excellent post-thaw survival, implantation, and pregnancy rates (129). Unfortunately, adult women with hematologic malignancies often encounter distinct clinical barriers that limit the feasibility of oocyte or embryo cryopreservation. The aggressive and rapidly progressive nature of many hematologic cancers frequently necessitates the urgent initiation of cytotoxic chemotherapy, leaving insufficient time to complete the two to three weeks ovarian stimulation cycle required for oocyte maturation and embryo generation. In addition, in patients with hematologic disorders, oocyte retrieval, which requires transvaginal needle puncture, poses significant challenges, as underlying coagulopathies and immunosuppression substantially increase the risks of hemorrhage and infection (26, 69, 81, 130). Nevertheless, reports describe successful embryo cryopreservation in patients with leukemia (131). Notably, the first successful pregnancy in a woman with acute leukemia who had previously undergone HSCT was reported in 2014, demonstrating that embryo cryopreservation can be a feasible option in selected cases (132).

#### 1.5.2 Ovarian tissue cryopreservation (OTC)

Unlike embryo and oocyte cryopreservation, which require 2–3 weeks of ovarian stimulation to mature the follicles, OTC does not involve hormone stimulation and can therefore be performed immediately (53, 133). Thus, OTC is an emerging viable option for women who cannot delay treatment or receive hormonal stimulation, those undergoing risk-reducing salpingo-oophorectomy, and prepubertal girls. Despite the American Society of Clinical Oncology (ASCO)/the American Society for Reproductive Medicine (ASRM) guideline strongly recommending fertility preservation before gonadotoxic cancer treatment (134), OTC remains underutilized in pediatric patients, even after ASRM declared OTC non-experimental in 2019 (135).

Comparative outcome data show that OTC yields clinical pregnancy and live birth rates like oocyte and embryo cryopreservation in cancer patients, with live birth rates of 32.3% for ovarian tissue, 25.8% for oocyte, and 35.3% for embryo cryopreservation, and a lower miscarriage rate for ovarian tissue compared to embryo cryopreservation (136). In a retrospective cohort study involving 93 patients diagnosed with sarcoma or tumors of the bone marrow and lymphatic system, immature oocytes obtained during OTC from girls aged 0–25 years were evaluated. The mean age at menarche was defined as 12.2 years, and participants were categorized into pre-menarche and post-menarche groups. The results demonstrated that the pre-menarche group yielded significantly fewer mature oocytes after the *in-vitro* maturation (IVM) period compared with the post-menarche group. Furthermore, in the pre-menarche cohort, the percentage of oocytes reaching the MII stage increased from 3.1% at retrieval to 7.2% after 24 hours of maturation, whereas in the post-menarche group, MII maturation rates increased from 2.0% to 18.4% over the same period (137). In another retrospective cohort study, a significant positive correlation was observed between patient age, the number of oocytes retrieved, and IVM success rates. This study further demonstrated that even among pre-menarche patients, age remains a critical determinant of developmental competence. Overall maturation rates in children younger than 5 years were significantly lower than those in children aged 5–10 years. Taken together, although OTC remains the primary fertility preservation option for prepubertal girls, these findings suggest that oocytes retrieved from very young patients may lack the intrinsic developmental capacity to fully mature and acquire fertilization competence (138).

Fertility restoration after OTC can be achieved through ovarian tissue transplantation (OTT), an established technique that has resulted in several live births worldwide. However, in patients with acute leukemia, reimplantation carries a risk of disease relapse due to potential malignant contamination of the ovarian cortex (79, 98, 139). Published data on contamination risk are conflicting: one systematic review reported malignant cells in 39% of cryopreserved samples from patients with hematological malignancies (12/22 after prior chemotherapy, 10/22 chemotherapy-naïve) (140), whereas other studies highly sensitive real-time or quantitative RT-PCR detected minimal or no contamination in certain cohorts (e.g., only 1/58 cases, a CML patient) (23). More targeted analyses have revealed leukemic infiltration in a substantial proportion of cases when disease-specific molecular markers are available; for example, 2/6 CML patients and 7/12 ALL patients tested positive by RT-qPCR, and xenotransplantation of ALL-derived tissue into mice led to intraperitoneal leukemia in four cases (98). A cohort of 26 patients with leukemia showed no histologic contamination; however, PCR revealed leukemic signals in 9 of them (22).

**Figure 2:**
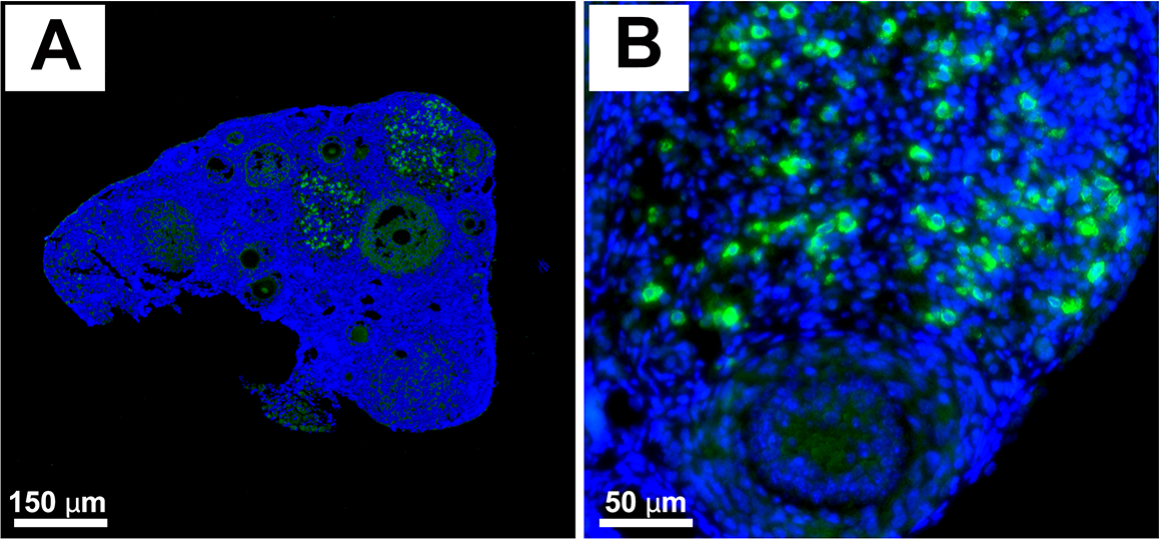
Leukemic cells penetrate the mouse ovary without damaging ovarian structure. Immunofluorescence images demonstrate GFP expression from 6-week-old female C57BL/6 mice injected with C1498-GFP mouse leukemic cells. The mice were given flank injections of 3.8 x 10^6^ cells on both sides, using 50% Dimethyl sulfoxide (DMSO) as the solvent, and they were euthanized after 7 days. **(A)** A high-magnification image of whole ovarian sections reveals GFP-positive leukemic cells present in the ovary. **(B)** Nuclei are counterstained with 4′,6-diamidino-2-phenylindole (DAPI; blue). GFP-positive leukemic cells are detected as green, fluorescent signals.

Recent experiments from our group’s study using a transgenic leukemic mouse model demonstrated that leukemic cells can infiltrate ovarian tissue and follicles, with a preferential localization in granulosa cells of antral follicles and in stromal compartments (99). Our additional allotransplantation experiments in a mouse model supported our previously published study. Immunofluorescence images from a mouse model injected with green fluorescent protein (GFP)-positive leukemic cells show that leukemic cells can infiltrate both ovarian tissue and follicles (see **Figure 2**). In this experiment, six-week-old female C57BL/6 mice were injected with 3.8 x 10^6^ C1498-GFP mouse leukemic cells in both flanks and then euthanized after seven days to collect the ovaries. The study reveals that leukemic cells can infiltrate the mouse ovary without causing obvious structural damage. GFP-labeled leukemic cells are interspersed among ovarian cells, as confirmed by their localization within the ovary. These results indicate that leukemic cells can penetrate ovarian tissue and establish residence without immediately disrupting its histological integrity. These findings highlight that relying solely on PCR-based approaches to detect leukemic cell infiltration may be insufficient. Therefore, prior to ovarian tissue cryopreservation, a portion of the harvested tissue should undergo careful pathological evaluation. A combination of histological assessment, immunohistochemistry, and disease-specific molecular analyses, such as RT-qPCR, is recommended to minimize the risk of reintroducing malignant cells during fertility-restoration procedures, particularly in patients who have undergone chemotherapy.

Researchers are exploring various experimental approaches to eliminate malignant contamination from ovarian tissue. These include ex vivo purging techniques that use targeted antibodies, chemotherapeutic agents, or selective inhibitors to eradicate leukemic cells while preserving follicular viability. Another promising strategy is the isolation and *in vitro* maturation (IVM) of follicles or oocytes from ovarian tissue, which could eliminate the need for reimplanting tissue altogether. Advances in artificial ovary systems and three-dimensional biomaterial scaffolds further enhance the possibility of transplanting isolated follicles into a controlled, leukemia-free environment. Xenotransplantation models have been used as functional assays to evaluate the presence of residual malignant cells, providing an important layer of safety before clinical application. A more experimental approach involves using female germ cells derived from pluripotent stem cells, enabling oocyte generation without using ovarian tissue (141). Although these strategies are still largely in the investigational stage, they are significant steps toward enhancing the safety of fertility restoration in leukemia patients. Ongoing refinement and validation of these approaches will be crucial for facilitating broader clinical application of OTT while minimizing the risk of disease recurrence.

In addition to established fertility preservation approaches, such as oocyte and embryo cryopreservation and ovarian tissue cryopreservation, several adjunctive strategies have been investigated to mitigate treatment-induced ovarian damage. Among these, the administration of gonadotropin-releasing hormone (GnRH) agonists before or during chemotherapy has been proposed as a potential protective intervention. It has been hypothesized that GnRH agonists may exert ovarian protection through both direct and indirect mechanisms, including suppression of gonadotropin secretion and possible modulation of intraovarian signaling pathways. However, the precise mechanisms underlying their protective effects remain incompletely understood. Although clinical studies have yielded mixed results, GnRH agonists are currently used in selected clinical contexts. However, their overall efficacy remains a subject of ongoing debate (142–144). Other approaches include ovarian shielding during pelvic radiotherapy, which aims to reduce radiation exposure and preserve ovarian reserve. However, its effectiveness remains questionable due to frequent inaccuracies in shield placement and significant anatomical variability in ovarian position, particularly in children and with changes in bladder filling. Moreover, emerging evidence suggests that even correctly positioned shields may be ineffective in preventing ovarian damage, raising concerns about their routine clinical use (145, 146). In addition, increasing attention has been directed toward pharmacological agents that prevent chemotherapy-induced follicle loss by targeting key molecular pathways involved in the DNA damage response and apoptosis. These approaches include inhibitors of DNA damage signaling components, such as CHK2 inhibitors (63, 108). However, most of these strategies remain at the preclinical stage and have not yet advanced to clinical application.

#### 1.5.3 Leukemia-related conditions affecting the safety of procedures

In patients with leukemia, hematologic abnormalities related to the disease and its treatment, especially thrombocytopenia and neutropenia, are critical factors influencing the safety of reproductive procedures such as transvaginal oocyte retrieval.

Thrombocytopenia, which is often caused by bone marrow infiltration or myelosuppressive chemotherapy, significantly heightens the risk of bleeding during procedures. Oocyte retrieval involves using transvaginal ultrasound to guide a needle into the ovarian cortex, requiring adequate platelet levels to reduce the risk of hemorrhagic complications. In clinical practice, platelet transfusions are frequently administered before the procedure to ensure that platelet counts reach a safe threshold. According to the American Academy of Family Physicians, prior to invasive procedures, platelet count should be above 40,000-50,000/µL. The American Society of Clinical Oncology and the American Association of Blood Blanks recommend a minimum platelet count of 50,000/µL prior to major surgery (147–149). Shigematsu et al. reported one case report at the Technique and Technology 2^nd^ Congress of the ASFP & FERTIPROTECT 2018 (5^th^ Annual Conference of the FPSI). They discussed three examples of oocyte cryopreservation in patients with thrombocytopenia secondary to hematological disorders. They transfused patients with platelets below 50,000/µL and were able to perform egg retrieval once their platelets were above 50,000/µL.

Neutropenia complicates procedural planning by increasing the risk of infection. The transvaginal route carries an inherent risk of microbial contamination, and in immunocompromised patients, even minor breaches in mucosal integrity can lead to severe or systemic infections. Therefore, it is essential to implement prophylactic antibiotics, maintain strict aseptic techniques, and carefully select patients to mitigate these risks (150). In cases of profound neutropenia, clinicians may choose to delay oocyte retrieval when possible or explore alternative fertility preservation strategies (119).

In addition to hematologic factors, it is essential to consider the overall clinical status of leukemia patients, including the burden of the disease, the urgency of starting treatment, and any concurrent complications. For many patients with acute leukemia, the immediate need for chemotherapy may prevent the necessary time for ovarian stimulation, which further limits the possibility of oocyte retrieval. These considerations highlight the importance of a multidisciplinary approach that involves oncologists, reproductive endocrinologists, and hematologists. This collaboration is crucial to balance procedural safety with the goal of preserving fertility.

### 1.6 Future direction

Remarkable advances in leukemia diagnosis and treatment have transformed survival outcomes across all major subtypes, particularly in children, adolescents, and young adults. As cure rates continue to rise, long-term survivorship issues, most notably fertility preservation, have emerged as critical components of comprehensive leukemia care. This review highlights that female reproductive health is uniquely vulnerable to both the systemic nature of leukemia and the gonadotoxic effects of its treatments. The ovary, which has a finite and nonrenewable pool of primordial follicles, is highly sensitive to gonadotoxic treatment such as chemotherapeutic agents, radiotherapy, and conditioning regimens used for HSCT, placing female patients at substantial risk of diminished ovarian reserve, POI, and infertility.

Beyond treatment-related toxicity, accumulating clinical and experimental evidence, including findings from mouse models, indicates that leukemic cells can directly infiltrate ovarian tissue and follicles, thereby altering the ovarian microenvironment through inflammatory and endocrine-disruptive mechanisms. These findings raise important biological and clinical concerns, particularly in the context of fertility preservation strategies that involve ovarian tissue harvesting, storage, or transplantation. The risk of malignant contamination underscores the need for rigorous screening and the continued development of safer, leukemia-specific fertility-restoration techniques.

Fertility preservation options for female leukemia patients have expanded significantly and now include oocyte and embryo cryopreservation, OTC, and emerging pharmacologic protective strategies. However, each approach carries distinct limitations related to patient age, pubertal status, treatment urgency, and disease-specific risks. Notably, prepubertal girls and patients requiring immediate therapy remain the most challenging populations, emphasizing the importance of early referral, multidisciplinary counseling, and individualized decision-making.

Looking forward, critical gaps remain in our understanding of leukemia-ovary interactions, long-term reproductive outcomes following modern targeted therapies, and optimal strategies to eliminate malignant contamination while preserving fertility. Addressing these gaps will require coordinated efforts among oncologists, reproductive specialists, and basic scientists. Ultimately, integrating fertility preservation into standard leukemia care is essential to improving not only survival, but also the long-term quality of life for female leukemia survivors.
